# The effect of fiber supplementation on the prevention of diarrhea in hospitalized patients receiving enteral nutrition: A meta-analysis of randomized controlled trials with the GRADE assessment

**DOI:** 10.3389/fnut.2022.1008464

**Published:** 2022-11-25

**Authors:** Apichat Kaewdech, Pimsiri Sripongpun, Panu Wetwittayakhlang, Chaitong Churuangsuk

**Affiliations:** ^1^Gastroenterology and Hepatology Unit, Division of Internal Medicine, Faculty of Medicine, Prince of Songkla University, Hat Yai, Thailand; ^2^Clinical Nutrition and Obesity Medicine Unit, Division of Internal Medicine, Faculty of Medicine, Prince of Songkla University, Hat Yai, Thailand

**Keywords:** nosocomial diarrhea, dietary fiber (DF), soluble fiber, psyllium, guar gum (GG), tube feeding, enteral nutrition

## Abstract

**Introduction:**

Enteral nutrition (EN) in hospitalized patients has several advantages. However, post-feeding diarrhea occurs frequently and has been linked to negative outcomes. The EN formula itself may have an impact on how diarrhea develops, and fiber supplements may theoretically help patients experience less diarrhea. This study aimed to thoroughly evaluate whether adding fiber to EN decreases the likelihood of developing diarrhea and whether different types of fibers pose different effects on diarrhea (PROSPERO CRD 42021279971).

**Methods:**

We conducted a meta-analysis on fiber supplementation in hospitalized adult patients receiving EN. We thoroughly searched PubMed, Medline, Embase, Scopus, Web of Science, CENTRAL, and ClinicalTrials.gov databases from inception to 1 September 2022. Only randomized controlled trials (RCTs) were included. Pooled results on the incidence of diarrhea were calculated using a random-effects model. The Grading of Recommendations, Assessment, Development, and Evaluations (GRADE) approach was applied. Only fiber types from soy polysaccharides (*n* = 4), psyllium (*n* = 3), mixed soluble/insoluble fiber (mixed fiber, *n* = 3), pectin (*n* = 2), and partially hydrolyzed guar gum (PHGG, *n* = 2) were examined in the sensitivity analysis.

**Results:**

Among the 4,469 titles found, a total of 16 RCTs were included. Overall, compared to fiber-free formulas, fiber supplementation reduced the occurrence of diarrhea in patients receiving EN by 36% (pooled risk ratio [RR] of 0.64 [95% confidence interval (CI): 0.49–0.82, *p* = 0.005; *I*^2^ = 45%]), with GRADE showing the evidence of moderate certainty. Only mixed fiber and PHGG significantly decreased the incidence of diarrhea according to the sensitivity analyses for fiber types (RR 0.54, 95%CI: 0.39–0.75, *I*^2^ = 0% and RR 0.47, 95%CI: 0.27–0.83, *I*^2^ = 0%, respectively). The results for the remaining fiber types were unclear.

**Conclusion:**

According to a meta-analysis, fiber supplements help lessen post-feeding diarrhea in hospitalized patients receiving EN. However, not all fiber types produced successful outcomes. Diarrhea was significantly reduced by PHGG and mixed soluble/insoluble fiber.

**Systematic review registration:**

https://www.crd.york.ac.uk/PROSPERO/display_record.php?RecordID=279971, identifier: PROSPERO CRD 42021279971.

## Introduction

Enteral nutrition (EN), a form of nutritional support delivered *via* the gastrointestinal tract, is preferred for hospitalized patients whose caloric and nutritional requirements cannot be adequately met by oral intake. EN has been proven to offer several benefits in such patients over parenteral nutrition, e.g., the maintenance of gut mucosal integrity, the reduction of bacterial translocation from the gut lumen to the blood stream, and the prevention of infection. Nonetheless, some gastrointestinal problems may occur in patients receiving EN. Diarrhea is one of the common conditions encountered, as observed in 29–39% of enterally fed patients (1–3), and can lead to unfavorable sequelae, such as volume and electrolyte disturbances, perianal dermatitis, and a longer duration of hospital stay (3–5).

Dietary fibers are parts of carbohydrates derived from plant cell wall components, which are neither digested nor absorbed in the small intestine. They have a degree of polymerization of ≥10 monomeric units, as defined by the World Health Organization (WHO), or three or more monomeric units, as specified by the European Food Safety Authority and by the US Food and Drug Administration (6). There are a variety of dietary fibers with different physiochemical characteristics. Dietary fibers consist of water-soluble and water-insoluble fibers. Soluble fibers, such as soy polysaccharides, psyllium, partially hydrolyzed guar gum (PHGG), pectin, banana flakes, Shen jia, and polydextrose, have been demonstrated to improve the regularity of bowel movement due to the luminal water-holding property of fibers to form bulky, soft, and easy-to-pass stools (7). In addition to improving regularity, insoluble fibers (e.g., wheat bran) can stimulate water and mucous secretion by irritating the large bowel mucosa (7).

In terms of tube-feeding diarrhea, several mechanisms proposed that dietary fiber supplementation in EN may yield a benefit in reducing the occurrence of diarrhea, e.g., increased viscosity of the stool content leading to bulk formation, prolongation of intestinal transit time, fermentability to produce short-chain fatty acids (SCFA), and exertion of several positive effects on colonocytes and colonic microbiota (6, 8–11). In the present meta-analysis, we aimed to systematically review the evidence from randomized controlled studies evaluating dietary fiber supplementation in the prevention of diarrhea in hospitalized patients requiring tube feeding.

## Methods

This systematic review and meta-analysis was conducted following a protocol registered in PROSPERO (CRD42021279971) and reported in accordance with the Preferred Reporting Items of Systematic Reviews and Meta-Analyses (PRISMA) guideline (12).

### Search and information sources

We systematically searched the Web of Science Core Collection, PubMed, Medline (OVID), Embase (OVID), and Scopus databases from inception to 1 September 2022. Cochrane Central Register of Controlled Trials (CENTRAL) and ClinicalTrials.gov were also searched for the trial registry. We also searched the reference lists of included full texts for additional articles. The search was limited to adult patients. No language limit was applied.

Search terms as free texts and MeSH terms related to “tube feeding” or “enteral nutrition,” “fiber,” and “diarrhea” or “bowel movement” were used. The following fibers reported in the literature were also used as search terms: inulin, psyllium, fructooligosaccharides (FOS), oligofructose, oligosaccharides, wheat brans, soy polysaccharides, lignin, and resistant starch. The full search strategy is available in Supplementary Table S1.

### Eligibility criteria

Studies were included if they were randomized controlled trials (RCTs), comparing fiber supplementation or fiber-enriched EN formula (any fiber type) with fiber-free EN formula and reporting the incidence/event outcome of diarrhea. Study participants were adults (aged ≥18 years old) and hospitalized in the intensive care unit (ICU) or non-ICU, receiving EN support with or without parenteral nutrition. Studies were excluded if there was no control arm or if patients received EN of < 1,000 kcal/day.

### Study selection and data extraction

All searched records were exported to EndNote (EndNote X8, Thomson Reuters, NY, USA) and deduplicated. Two reviewers (PS and AK) independently screened the titles and abstracts of eligible papers. When there were disagreements between the two reviewers, a consensus was reached out and the third reviewer (CC or PW) was consulted. Data extraction was performed independently by PS and AK. CC was consulted when there were any problems related to data extraction. Data extraction was performed for authors, years, title, population characteristics and setting, fiber types and dosage, the duration of EN, energy intake, the definition of diarrhea and/or methods for measuring diarrhea, and the incidence or event rate of diarrhea.

### Risk of bias (quality) assessment

Two reviewers (PS and AK) independently assessed the risk of bias among the included papers using the Cochrane Risk of Bias 2.0 tool (RoB2) for RCTs (13). The RoB2 comprises five domains: bias arising from the randomization process, bias due to deviations from intended interventions, bias due to lack of outcome data, bias in outcome measurement, and bias in the selection of the reported result.

### Data synthesis

The incidence or event rate of diarrhea was pooled using the Mantel–Haenszel methods (for the binary outcome) and presented as risk ratio (RR) and 95% confidence interval (CI). A random-effects model was applied for pooled estimates due to the increased chance of high heterogeneity among included studies. The *I*^2^ statistic was used to assess heterogeneity. A heterogeneity of >50% will be judged as high, with a *p*-value of < 0.10 for significance. Sources of heterogeneity were explored by subgroup analysis/sensitivity analysis. Pre-priori subgroup analyses were planned for the non-ICU and ICU settings, low vs. high RoB studies, and fiber types. All statistical analyses were performed using R software, version 4.1.0 (R Foundation, Austria) with the Metafor package. A two-sided *p*-value of < 0.05 was considered statistically significant.

Publication bias was analyzed using the funnel plot and Egger's test for funnel plot asymmetry. The Grading of Recommendations, Assessment, Development, and Evaluations (GRADE) system was applied for pooled results, which comprises types of study, quality of methodology, consistency of outcomes, directness, effect size, and publication bias (14, 15).

## Results

**Figure 1:**
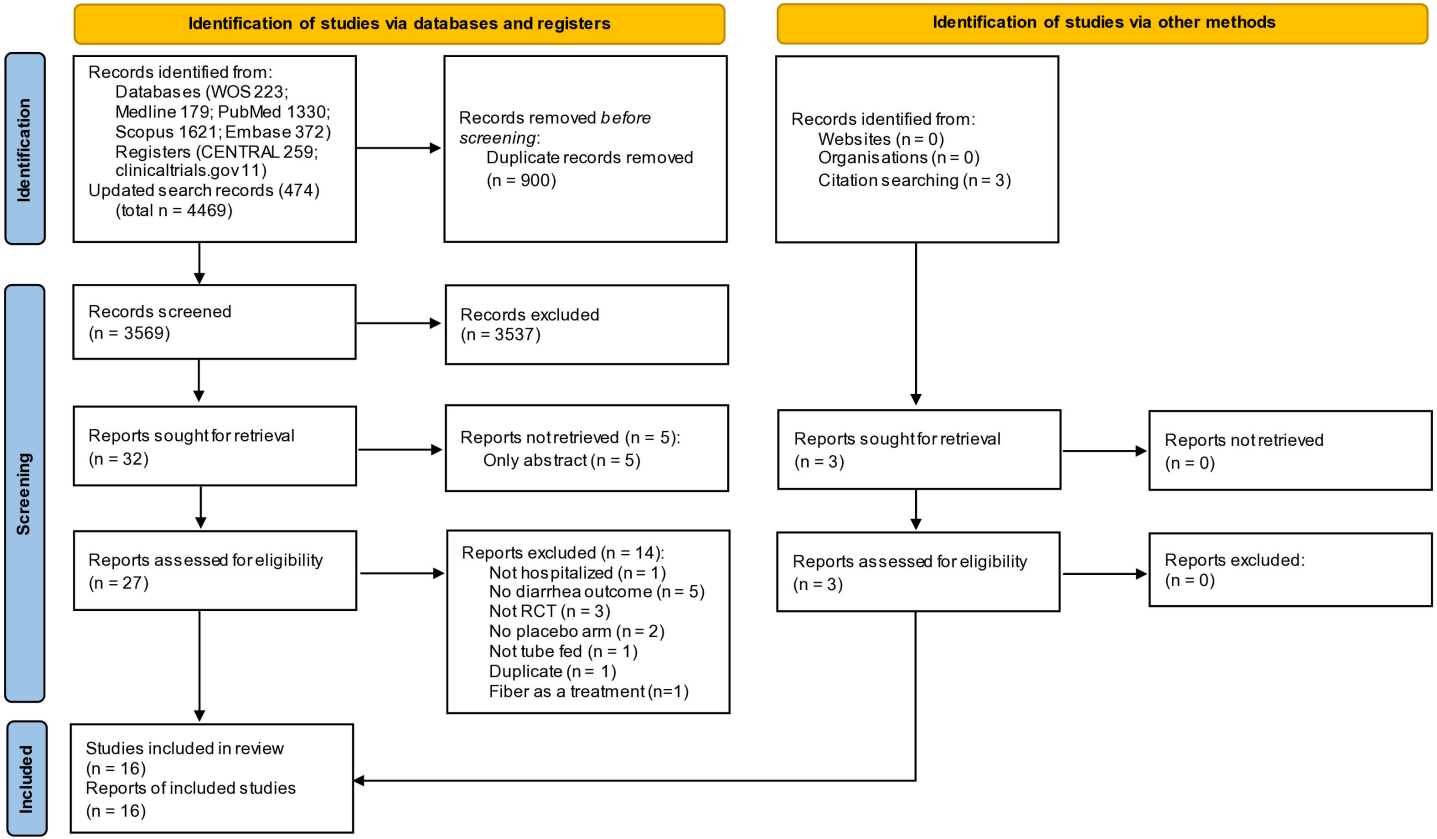
Preferred reporting items of systematic reviews and meta-analyses (PRISMA) flow diagram.

A total of 4,469 records were retrieved from the literature search, and three records were from additional sources. After removing duplicates, two reviewers independently screened 3,569 records for titles and abstracts, resulting in 27 full texts that were assessed for eligibility criteria. There were 17 RCTs evaluating the role of fiber supplementation on the outcomes of diarrhea in hospitalized tube-fed patients. Of these 17, only one RCT explored the role of fiber (banana flakes) vs. routine medical treatment in patients who already developed diarrhea (16), and the remaining 16 RCTs were conducted in a general tube-feeding setting to evaluate the occurrence of diarrhea. The last 16 RCTs were included in the present meta-analysis (Figure 1). Excluded full texts are shown in Supplementary Table S2, with reasons.

### Study characteristics and bias of included RCTs

Most RCTs were conducted in ICU patients (*n* = 11) (17–27), followed by postoperative patients (*n* = 3) (28–30) and hospitalized patients (*n* = 2) (31, 32). The majority of RCTs investigated fiber supplements in EN using soy polysaccharides (*n* = 4), followed by mixed soluble/insoluble fiber (*n* = 3), psyllium (*n* = 3), PHGG (*n* = 2), pectin (*n* = 2), Shen jia (*n* = 1), and polydextrose (*n* = 1). The median duration of fiber supplementation was 10 days, ranging from 5 to 21 days. Diarrhea was defined based on diarrhea score, number of bowel movements per day, and Bristol or King stool chart (Table 1).

**Table 1:** Characteristics of included randomized controlled trials (RCTs).

**Author**	**Intervention**			**Control**			**Setting**	**Outcome time**	**Diarrhea definition**	**Route of EN**	**Fiber dosage**
	**Fiber types**	**No. diarrhea**	**No. total**	**Control**	**No. diarrhea**	**No. total**					
**ICU setting**											
Frankenfield and Beyer (18)	soy polysaccharide	3	9	Ensure	4	9	ICU head injury	6 days	1/3 criteria	NG	14 gm/L
Dobb and Towler (19)	soy polysaccharide	16	45	Ensure	13	46	ICU	18 days	diarrhea score >12	NG/PEG	21 gm/L
Tuncay et al. (26)	soy polysaccharide	2	23	Osmolite	13	23	Neurological ICU	21 days	not defined	NG/PEG	14.4 gm/L
Chittawatanarat et al. (23)	mixed soluble/insoluble	4	17	Nutren Optimum	8	17	ICU	14 days	diarrhea score >12	no defined	15.1 gm/L
Yagmurdur and Leblebici (24)	mixed soluble/insoluble	22	60	Nutrison	38	60	MICU	5 days	diarrhea score >12	NG	15 gm/L
Hart and Dobb (17)	psyllium	19	35	Osmolite	19	33	ICU	18 days	diarrhea score >12	NG	7 gm/d
Belknap et al. (20)	psyllium hydrophilic mucilloid	8	37	Ensure / Osmolite	7	23	Medical and surgical ICU	7 days	≥3 bowel movement a day	NG/PEG	14 gm/d
Schultz et al. (21)	pectin	4	11	Osmolite	1	11	ICU	8 days	diarrhea score >12	feeding tube	1.07 g/d
Xi et al. (25)	pectin	7	62	Peptisorb	16	63	ICU	6 days	not defined	NJ	24 gm/day
Spapen et al. (22)	PHGG	6	13	no label	11	12	Medical ICU	21 days	diarrhea score >12	NG	22 gm/L
Chen et al. (27)	polydextrose	2	24	no label	9	22	ICU	7 days	≥3 bowel movement a day	NJ	20 gm/d
**Non-ICU**											
de Kruif and Vos (28)	soy polysaccharide	8	30	Osmolite	14	30	post-operative patients	5 days	diarrhea score >6 x 2 days	NG/NJ	20 gm/L
Jakobsen et al. (31)	mixed soluble/insoluble	5	26	no label	12	25	hospitalized patients	14 days	Daily defecation score >15	NG/PEG	15 gm/L
Lertpipopmetha et al. (32)	psyllium	18	42	Blendera	13	41	hospitalized medical patients	10 days	King's stool chart ≥15	NG	15.2 gm/L
Homann et al. (29)	PHGG	2	15	Nutrodrip	6	15	upper gastrointestinal surgery	10 days	≥3 bowel movements a day	jejunostomy	20 gm/L
Zhao et al. (30)	Shen jia	12	40	no label	24	40	gastric cancer post distal gastrectomy	7 days	King's stool chart ≥15	NJ	30 gm/day

ICU, intensive care unit; PHGG, partially hydrolyzed guar gum; EN, enteral nutrition; NG, naso-gastric; PEG, percutaneous endoscopic gastrostomy; NJ, naso-jejunostomy.

More than half of the included RCTs (*n* = 9) are at high risk of bias (Table 2). Of these, eight RCTs did not report information about whether outcome assessors were aware of the intervention received by study participants. For such reasons, the assessment of the outcome could have been influenced by knowledge of the intervention received. The funnel plot of 16 RCTs shows no publication bias, with Egger's *p*-value being 0.216 (Supplementary Figure S1).

**Table 2:** Risk of bias of included RCTs.

**References**	**Experimental**	**Randomization process**	**Deviations from intended interventions**	**Missing outcome data**	**Measurement of the outcome**	**Selection of the reported result**	**Overall bas**
Hart and Dobb (17)	Psyllium	Some concerns	Low	Low	High	Low	High
Frankenfield and Beyer (18)	Soy polysaccharide	Some concerns	Low	Low	High	High	High
Dobb and Towler (19)	Soy polysaccharide	Low	Low	Low	High	Low	High
de Kruif and Vos (28)	Soy polysaccharide	Low	Low	Low	High	Low	High
Homann et al. (29)	PHGG	Some concerns	Low	Low	Low	Low	Some concerns
Belknap et al. (20)	Psyllium	Low	Some concerns	Low	High	Low	High
Schultz et al. (21)	Pectin	Some concerns	Low	Low	Low	Low	Some concerns
Spapen et al. (22)	PHGG	Some concerns	Low	High	Low	Low	High
Chittawatanarat et al. (23)	Mixed soluble/insoluble	Low	Low	Low	Low	Low	Low
Yagmurdur and Leblebici (24)	Mixed soluble/insoluble	Low	Low	Low	Low	Low	Low
Jakobsen et al. (31)	Mixed soluble/insoluble	Low	Low	Low	Low	Low	Low
Xi et al. (25)	Pectin	Some concerns	Low	High	High	Low	High
Zhao et al. (30)	Shen jia	Low	Low	Low	High	Low	High
Tuncay et al. (26)	Soy polysaccharide	Some concerns	Low	High	High	Low	High
Lertpipopmetha et al. (32)	Psyllium	Low	Low	Low	Low	Low	Low
Chen et al. (27)	Polydextrose	Low	Low	Low	Low	Low	Low

PHGG, partially hydrolyzed guar gum.

### Effect of fiber supplementation on the incidence of diarrhea

**Figure 2:**
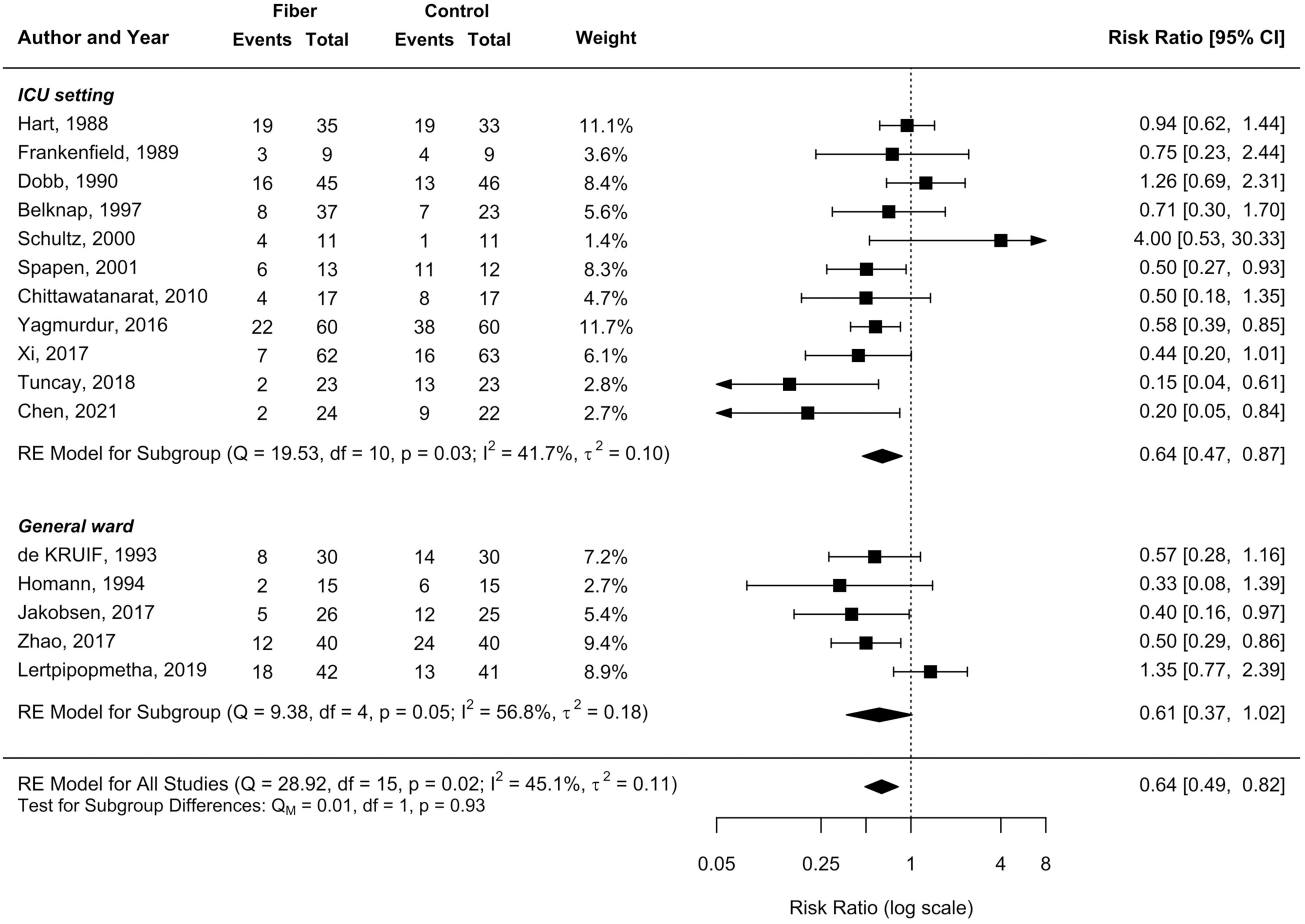
Effect of enteral nutrition (EN) containing fiber supplements on the incidence of diarrhea.

A meta-analysis of all 16 RCTs showed that fiber supplementation prevented the occurrence of diarrhea in hospitalized patients receiving EN by 36% compared to the fiber-free formula (pooled RR of 0.64 [95%CI: 0.49–0.82, *p* = 0.005]; *I*^2^ = 45.1%; Figure 2), with the GRADE assessment of moderate certainty (Table 3).

**Table 3:** Summary of findings with the grading of recommendations, assessment, development, and evaluations (GRADE) assessment.

**Summary of findings**
**Fiber in EN compared to non-fiber formula for prevention of diarrhea in patients with tube-feeding**						
**Patient or population:** prevention of diarrhea in tube-fed patients						
**Setting:** in hospital						
**Intervention:** fiber in EN						
**Comparison:** non-fiber formula						
**Outcome**	**Relative effect**	Anticipated absolute effects^*^(95% CI)			**Certainty**	**What happens**
**No of participants (studies)**	**(95% CI)**	**Without fiber**	**With fiber**	**Difference**		
Diarrhea	**RR 0.64**	44.3%	**28.3%**	**15.9% fewer**	⊕⊕⊕○	Fiber supplementation in enteral nutrition
No of participants: 959	(0.49 to 0.82)		(21.7 to 36.3)	(22.6 fewer to 8 fewer)	Moderate^a^, ^b^	likely reduces diarrhea in tube-fed hospitalized
(16 RCTs)^a^						patients

^*^The risk in the intervention group (95% CI) is based on the assumed risk in the comparison group and the relative effect of the intervention (95% CI).

EN, enteral nutrition; CI, confidence interval; RR, risk ratio.

GRADE working group grades of evidence.

High certainty: we are very confident that the true effect lies close to that of the estimate of the effect. Moderate certainty: we are moderately confident in the effect estimate: the true effect is likely to be close to the estimate of the effect, but there is a possibility that it is substantially different. Low certainty: our confidence in the effect estimate is limited: the true effect may be substantially different from the estimate of the effect. Very low certainty: we have very little confidence in the effect estimate: the true effect is likely to be substantially different from the estimate of effect.

^a^Subgroup analysis between “low” and “some concern or high" risk of bias shows a similar direction and magnitude of pooled results. We did not downgrade for this domain.

^b^Downgrade one level due to inconsistency. The pooled result shows moderate heterogeneity, with fiber type as a possible source of heterogeneity.

**Figure 3:**
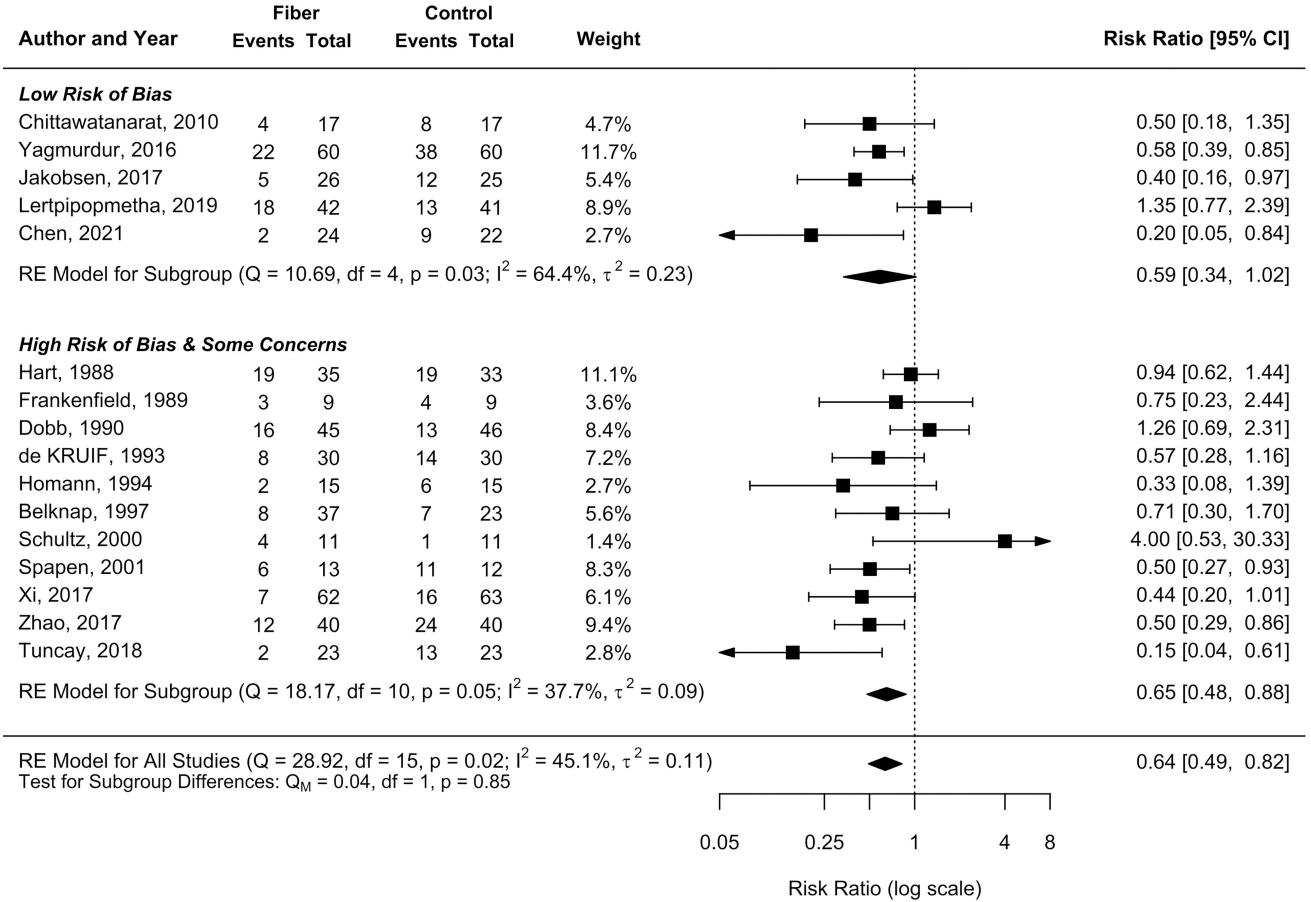
Subgroup by risk of bias on the effect of EN containing fiber supplements on the incidence of diarrhea.

Among the 11 RCTs conducted in the ICU setting, there was a 36% significant reduction in the incidence of diarrhea after fiber supplementation (pooled RR 0.64, 95%CI 0.47–0.87, *I*^2^ = 41.7%; Figure 2). In the non-ICU setting, a large effect size was observed in reducing the incidence of diarrhea (pooled RR 0.61, 95%CI 0.37–1.02), with high heterogeneity (*I*^2^ = 57%, *p* = 0.05; Figure 2). Similarly, the subgroup analysis between RCTs with a low risk of bias showed a large magnitude of effect size for the prevention of diarrhea (pooled RR 0.59, 95%CI 0.34–1.02), consistent with the pooled result of RCTs with some concerns or a high risk of bias (pooled RR 0.65, 95%CI 0.48–0.88; Figure 3).

**Figure 4:**
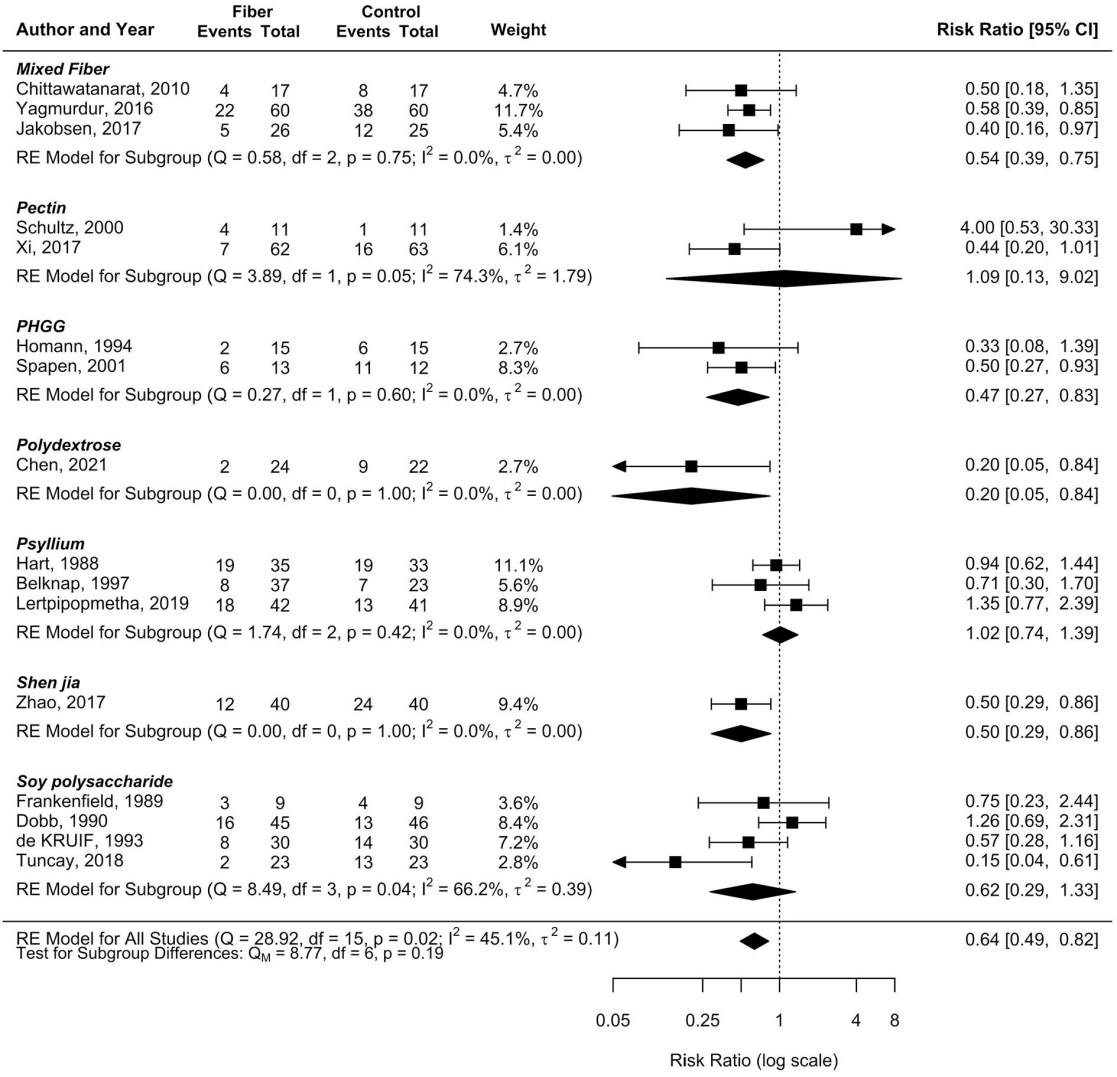
Effect of fiber types in EN on the incidence of diarrhea.

According to sensitivity analyses, we analyzed fiber types with at least two RCTs to explore whether fiber types posed different outcomes (Figure 4). These included soy polysaccharides (*n* = 4), psyllium (*n* = 3), mixed soluble/insoluble fiber (*n* = 3), pectin (*n* = 2), and PHGG (*n* = 2). There were reductions in post-feeding diarrhea in patients receiving EN containing mixed soluble/insoluble fiber and PHGG (pooled RR 0.54, 95%CI: 0.39–0.75, *I*^2^ = 0% and pooled RR 0.47, 95%CI: 0.27–0.83, *I*^2^ = 0%, respectively), while the remaining fiber types posed no benefits (Figure 4).

## Discussion

The current systematic review and meta-analysis examines the efficacy of fiber supplementation on the outcomes of diarrhea in hospitalized patients receiving tube feeding. We included only data from randomized control studies. Overall, fiber supplementation was significantly associated with a reduced risk of developing diarrhea in such patients (pooled RR of 0.64 [95% CI: 0.49–0.82, *p* = 0.005]), but with moderate heterogeneity (*I*^2^ = 45.1%).

We further performed sensitivity analyses to identify plausible explanations for the heterogeneity of the results. As determined *a priori*, sensitivity analyses regarding patient settings (ICU vs. non-ICU), low vs. high RoB studies, and fiber types were conducted. Regarding the patient settings, the benefit of fiber supplementation was observed in both critically ill patients and patients admitted to general medical or surgical wards, with similarly pooled RRs of 0.64 and 0.61, respectively. Although only patients in the ICU group reached a statistically significant level, patients in the non-ICU setting had a 95% CI slightly above 1 (95%CI: 0.37–1.02). Moreover, moderate heterogeneity persisted in both ICU and non-ICU patients.

Similar results were observed when we conducted sensitivity analyses of studies with low and high RoB; the effect sizes of fiber supplementation in reducing the risk of occurrence of diarrhea were allied and reached a statistically significant in the high RoB study group with moderate heterogeneity, while the upper level of 95%CI in the low RoB study group was only 1.02, with high heterogeneity.

Interesting findings were observed in sensitivity analyses by fiber type. Soy polysaccharides, the most frequently studied fiber in the literature, resulted in a non-significant reduction in the outcome of diarrhea, with a high degree of heterogeneity. Psyllium and mixed soluble/insoluble fiber were evaluated in the following order, and each had three RCT data. Intriguingly, the pooled RR of both types of fiber showed no heterogeneity (*I*^2^ = 0%) when sensitivity analyses were executed, and psyllium consistently showed no benefits in reducing the occurrence of diarrhea in patients receiving tube feeding, whereas mixed soluble/insoluble fiber significantly reduced the risk of developing diarrhea in such patients by 46% (RR 0.54 [95% CI: 0.39, 0.75]). The PHGG fiber type also showed a significant reduction in the incidence of diarrhea by 53% without heterogeneity. Compared with a recent meta-analysis by Cara et al. (33), mixed soluble/insoluble fiber did not reduce the incidence of diarrhea (RR 0.61 [95% CI: 0.37, 1.00]). However, such findings might be due to the high rate of diarrhea in a study by Schultz et al. (21).

To the best of our knowledge, this is the first study to show a novel finding of the significance of fiber types on the outcomes of diarrhea in hospitalized patients receiving EN. Our study results contradict a previous meta-analysis published in 2015, in which the benefit of fiber supplementation was observed only in non-critically ill patients and not in the ICU setting (34). Nevertheless, the current study results on the benefit of fiber supplementation in critically ill patients were consistent with a recent meta-analysis of dietary fiber in critical care patients published in 2021 (35). From our point of view, differences in the results between our meta-analysis and the prior meta-analysis by Kamarul Zaman et al. (34) might be due to differences in the study inclusion criteria, as we only included randomized control studies, and seven RCTs conducted after 2015 were added to our recent meta-analysis. Moreover, as shown in the aforementioned sensitivity analysis, the root cause of a variety of outcomes among RCTs might lay in the different types of fiber rather than in the critical care setting of patients.

Theoretically, soluble fibers have the beneficial properties of reducing diarrhea with their water-holding capacity and increasing gut transit time, and they can be fermented by colonic bacteria to produce SCFAs and stimulate the uptake of water and electrolytes in the colon (8, 9, 11). However, when it comes to the results of clinical studies, not all soluble fibers yielded the same benefit on the outcomes of diarrhea. This might be due to the diversity of physiochemical characteristics of each fiber type. The presence of either a soluble or insoluble fiber in the ileum can stimulate the ileal brake, resulting in decreased gastric emptying and increased small intestinal transit time, making the whole gut to be delayed (6). Despite being a soluble fiber, psyllium is considered to have moderate viscosity and low fermentability (6). Guar gum, on the other hand, owes the characteristics of medium to high viscosity and high fermentability (6). A higher degree of viscosity may result in increased stool volume and longer colonic transit time, and increased fermentability, as well as increased integrity of colonic tight junctions, may provide a better microbiota environment in the colon, together leading to a better outcome for some types of fiber over others. This benefit may further minimize patients' morbidity, length of hospital stay, investigation cost, and healthcare burden (3–5). Additionally, fiber supplementation is safe in hospitalized patients with stable hemodynamics (33). As such, our findings encourage healthcare professionals to recognize the beneficial effects of fiber supplementation in hospitalized patients receiving EN.

The strengths of our systematic review and meta-analysis are that we only included randomized controlled studies with a high-quality study design, from inception to the most recent timeframe, with over 700 patients from both critical and non-critical care settings, both surgical and medical patients. The source of heterogeneity can also be identified and minimized to the level of no heterogeneity in psyllium, PHGG, and mixed soluble/insoluble fiber subgroups using sensitivity analyses on fiber types. This novel finding and possible underlying mechanisms can be important in aiding the management of diarrhea in patients receiving EN in the future and for further studies.

Our meta-analysis also has limitations. There is a variation in the definition of diarrhea; some studies used scoring systems, while others counted the frequency of bowel movements or did not mention the definition of diarrhea in the study. This may influence the rates of occurrence of diarrhea in the included studies. Additionally, the fiber dosage varied; in some studies, the daily dosage of fiber was fixed in all patients in the fiber arm, whereas the fiber dosage administered to patients was dependent on the amount of calorie intake in a day in many studies, making an evaluation of the fiber dosage and the outcomes of diarrhea unattainable. Furthermore, there was a small number of participants in each fiber type; therefore, the power of performance assessment to determine the efficacy of different fiber types may be limited. Lastly, the variety of causes of critically ill patients could potentially affect the severity of post-feeding diarrhea, so further studies with a homogenous population should be conducted.

In conclusion, our recent systematic review and meta-analysis demonstrated a beneficial effect of fiber supplementation in minimizing diarrhea in hospitalized patients receiving tube feeding. However, not all fiber types yielded the same benefit; mixed soluble/insoluble fiber and PHGG are associated with a significant reduction in the risk of developing diarrhea, whereas studies on psyllium consistently showed no benefit over the fiber-free formula. For other types of fiber, no conclusion can be drawn at this time.

## Data availability statement

The original contributions presented in the study are included in the article/Supplementary material, further inquiries can be directed to the corresponding author.

## Author contributions

Protocol development: PS, PW, CC, and AK. Systematic literature search: PW and CC. Study selection and data extraction and risk of bias assessment: PS and AK. Data analysis and manuscript writing: PS and CC. Critical revision of the manuscript: AK, PS, and CC. All authors approved the final version of the manuscript.

## Conflict of interest

The authors declare that the research was conducted in the absence of any commercial or financial relationships that could be construed as a potential conflict of interest.

## Publisher's note

All claims expressed in this article are solely those of the authors and do not necessarily represent those of their affiliated organizations, or those of the publisher, the editors and the reviewers. Any product that may be evaluated in this article, or claim that may be made by its manufacturer, is not guaranteed or endorsed by the publisher.
