# Supplementary material for: The effect of fiber supplementation on the prevention of diarrhea in hospitalized patients receiving enteral nutrition: A meta-analysis of randomized controlled trials with the GRADE assessment
Source: Front Nutr. 2022 Nov 25;9:1008464. doi: 10.3389/fnut.2022.1008464 (PMC9733536; doi:10.3389/fnut.2022.1008464)
Supplement: Supplementary file 1 [file Data_Sheet_1.docx]

Supplementary Material

**Supplementary Table S1: Search strategy for PubMed**

| 1 | Tube-fed |
| --- | --- |
| 2 | Tube feeding |
| 3 | Enteral nutrition |
| 4 | Enteral feeding |
| 5 | Enteral alimentation |
| 6 | Nutritional support |
| 7 | Nutrition therapy |
| 8 | ((((((Tube-fed) OR (Tube feeding)) OR (Enteral nutrition)) OR (Enteral feeding)) OR (Enteral alimentation)) OR (Nutritional support)) OR (Nutrition therapy) |
| 9 | ((((((Tube-fed) OR (Tube feeding)) OR (Enteral nutrition)) OR (Enteral feeding)) OR (Enteral alimentation)) OR (Nutritional support)) OR (Nutrition therapy) |
| 10 | ((((((Tube-fed) OR (Tube feeding)) OR (Enteral nutrition)) OR (Enteral feeding)) OR (Enteral alimentation)) OR (Nutritional support)) OR (Nutrition therapy) |
| 11 | Fiber |
| 12 | Fiber-enriched |
| 13 | Fibre |
| 14 | inulin |
| 15 | Psyllium |
| 16 | FOS |
| 17 | Fructo-oligosaccharides |
| 18 | Oligofructose |
| 19 | oligosaccharides |
| 20 | Wheat bran |
| 21 | Soy polysaccharides |
| 22 | Lignin |
| 23 | Resistant starch |
| 24 | ((((((((((((Fiber) OR (Fiber-enriched)) OR (Fibre)) OR (inulin)) OR (Psyllium)) OR (FOS)) OR (Fructo-oligosaccharides)) OR (Oligofructose)) OR (oligosaccharides)) OR (Wheat bran)) OR (Soy polysaccharides)) OR (Lignin)) OR (Resistant starch) |
| 25 | ((((((((((((Fiber) OR (Fiber-enriched)) OR (Fibre)) OR (inulin)) OR (Psyllium)) OR (FOS)) OR (Fructo-oligosaccharides)) OR (Oligofructose)) OR (oligosaccharides)) OR (Wheat bran)) OR (Soy polysaccharides)) OR (Lignin)) OR (Resistant starch) |
| 26 | ((((((((((((Fiber) OR (Fiber-enriched)) OR (Fibre)) OR (inulin)) OR (Psyllium)) OR (FOS)) OR (Fructo-oligosaccharides)) OR (Oligofructose)) OR (oligosaccharides)) OR (Wheat bran)) OR (Soy polysaccharides)) OR (Lignin)) OR (Resistant starch) |
| 27 | Diarrhea |
| 28 | Diarrhoea |
| 29 | diarrheal |
| 30 | Bowel movement |
| 31 | Stool |
| 32 | Feces |
| 33 | Fecal |
| 34 | Stool form |
| 35 | Stool frequency |
| 36 | (((((((((Diarrhea) OR (Diarrhoea)) OR (diarrheal)) OR (Bowel movement)) OR (Stool)) OR (Feces)) OR (Fecal)) OR (Stool form)) OR (Stool frequency)) OR (Stool frequency) |
| 37 | (((((((((Diarrhea) OR (Diarrhoea)) OR (diarrheal)) OR (Bowel movement)) OR (Stool)) OR (Feces)) OR (Fecal)) OR (Stool form)) OR (Stool frequency)) OR (Stool frequency) |
| 38 | (((((((((Diarrhea) OR (Diarrhoea)) OR (diarrheal)) OR (Bowel movement)) OR (Stool)) OR (Feces)) OR (Fecal)) OR (Stool form)) OR (Stool frequency)) OR (Stool frequency) |
| 40 | ((((((((Tube-fed) OR (Tube feeding)) OR (Enteral nutrition)) OR (Enteral feeding)) OR (Enteral alimentation)) OR (Nutritional support)) OR (Nutrition therapy)) AND (((((((((((((Fiber) OR (Fiber-enriched)) OR (Fibre)) OR (inulin)) OR (Psyllium)) OR (FOS)) OR (Fructo-oligosaccharides)) OR (Oligofructose)) OR (oligosaccharides)) OR (Wheat bran)) OR (Soy polysaccharides)) OR (Lignin)) OR (Resistant starch))) AND ((((((((((Diarrhea) OR (Diarrhoea)) OR (diarrheal)) OR (Bowel movement)) OR (Stool)) OR (Feces)) OR (Fecal)) OR (Stool form)) OR (Stool frequency)) OR (Stool frequency)) |
| 41 | ((((((((Tube-fed) OR (Tube feeding)) OR (Enteral nutrition)) OR (Enteral feeding)) OR (Enteral alimentation)) OR (Nutritional support)) OR (Nutrition therapy)) AND (((((((((((((Fiber) OR (Fiber-enriched)) OR (Fibre)) OR (inulin)) OR (Psyllium)) OR (FOS)) OR (Fructo-oligosaccharides)) OR (Oligofructose)) OR (oligosaccharides)) OR (Wheat bran)) OR (Soy polysaccharides)) OR (Lignin)) OR (Resistant starch))) AND ((((((((((Diarrhea) OR (Diarrhoea)) OR (diarrheal)) OR (Bowel movement)) OR (Stool)) OR (Feces)) OR (Fecal)) OR (Stool form)) OR (Stool frequency)) OR (Stool frequency)) |
| 42 | ((((((((Tube-fed) OR (Tube feeding)) OR (Enteral nutrition)) OR (Enteral feeding)) OR (Enteral alimentation)) OR (Nutritional support)) OR (Nutrition therapy)) AND (((((((((((((Fiber) OR (Fiber-enriched)) OR (Fibre)) OR (inulin)) OR (Psyllium)) OR (FOS)) OR (Fructo-oligosaccharides)) OR (Oligofructose)) OR (oligosaccharides)) OR (Wheat bran)) OR (Soy polysaccharides)) OR (Lignin)) OR (Resistant starch))) AND ((((((((((Diarrhea) OR (Diarrhoea)) OR (diarrheal)) OR (Bowel movement)) OR (Stool)) OR (Feces)) OR (Fecal)) OR (Stool form)) OR (Stool frequency)) OR (Stool frequency)) |
| 43 | enteral nutrition[MeSH Terms] |
| 44 | ((((((((Tube-fed) OR (Tube feeding)) OR (Enteral nutrition)) OR (Enteral feeding)) OR (Enteral alimentation)) OR (Nutritional support)) OR (Nutrition therapy)) AND (((((((((((((Fiber) OR (Fiber-enriched)) OR (Fibre)) OR (inulin)) OR (Psyllium)) OR (FOS)) OR (Fructo-oligosaccharides)) OR (Oligofructose)) OR (oligosaccharides)) OR (Wheat bran)) OR (Soy polysaccharides)) OR (Lignin)) OR (Resistant starch))) AND ((((((((((Diarrhea) OR (Diarrhoea)) OR (diarrheal)) OR (Bowel movement)) OR (Stool)) OR (Feces)) OR (Fecal)) OR (Stool form)) OR (Stool frequency)) OR (Stool frequency)) |
| 45 | ((((((((Tube-fed) OR (Tube feeding)) OR (Enteral nutrition)) OR (Enteral feeding)) OR (Enteral alimentation)) OR (Nutritional support)) OR (Nutrition therapy)) AND (((((((((((((Fiber) OR (Fiber-enriched)) OR (Fibre)) OR (inulin)) OR (Psyllium)) OR (FOS)) OR (Fructo-oligosaccharides)) OR (Oligofructose)) OR (oligosaccharides)) OR (Wheat bran)) OR (Soy polysaccharides)) OR (Lignin)) OR (Resistant starch))) AND ((((((((((Diarrhea) OR (Diarrhoea)) OR (diarrheal)) OR (Bowel movement)) OR (Stool)) OR (Feces)) OR (Fecal)) OR (Stool form)) OR (Stool frequency)) OR (Stool frequency)) |
| 46 | ((((((((Tube-fed) OR (Tube feeding)) OR (Enteral nutrition)) OR (Enteral feeding)) OR (Enteral alimentation)) OR (Nutritional support)) OR (Nutrition therapy)) AND (((((((((((((Fiber) OR (Fiber-enriched)) OR (Fibre)) OR (inulin)) OR (Psyllium)) OR (FOS)) OR (Fructo-oligosaccharides)) OR (Oligofructose)) OR (oligosaccharides)) OR (Wheat bran)) OR (Soy polysaccharides)) OR (Lignin)) OR (Resistant starch))) AND ((((((((((Diarrhea) OR (Diarrhoea)) OR (diarrheal)) OR (Bowel movement)) OR (Stool)) OR (Feces)) OR (Fecal)) OR (Stool form)) OR (Stool frequency)) OR (Stool frequency)) |
| 47 | ((((((((Tube-fed) OR (Tube feeding)) OR (Enteral nutrition)) OR (Enteral feeding)) OR (Enteral alimentation)) OR (Nutritional support)) OR (Nutrition therapy)) AND (((((((((((((Fiber) OR (Fiber-enriched)) OR (Fibre)) OR (inulin)) OR (Psyllium)) OR (FOS)) OR (Fructo-oligosaccharides)) OR (Oligofructose)) OR (oligosaccharides)) OR (Wheat bran)) OR (Soy polysaccharides)) OR (Lignin)) OR (Resistant starch))) AND ((((((((((Diarrhea) OR (Diarrhoea)) OR (diarrheal)) OR (Bowel movement)) OR (Stool)) OR (Feces)) OR (Fecal)) OR (Stool form)) OR (Stool frequency)) OR (Stool frequency)) |
| 48 | ((((((((Tube-fed) OR (Tube feeding)) OR (Enteral nutrition)) OR (Enteral feeding)) OR (Enteral alimentation)) OR (Nutritional support)) OR (Nutrition therapy)) AND (((((((((((((Fiber) OR (Fiber-enriched)) OR (Fibre)) OR (inulin)) OR (Psyllium)) OR (FOS)) OR (Fructo-oligosaccharides)) OR (Oligofructose)) OR (oligosaccharides)) OR (Wheat bran)) OR (Soy polysaccharides)) OR (Lignin)) OR (Resistant starch))) AND ((((((((((Diarrhea) OR (Diarrhoea)) OR (diarrheal)) OR (Bowel movement)) OR (Stool)) OR (Feces)) OR (Fecal)) OR (Stool form)) OR (Stool frequency)) OR (Stool frequency)) |
| 49 | ((((((((Tube-fed) OR (Tube feeding)) OR (Enteral nutrition)) OR (Enteral feeding)) OR (Enteral alimentation)) OR (Nutritional support)) OR (Nutrition therapy)) AND (((((((((((((Fiber) OR (Fiber-enriched)) OR (Fibre)) OR (inulin)) OR (Psyllium)) OR (FOS)) OR (Fructo-oligosaccharides)) OR (Oligofructose)) OR (oligosaccharides)) OR (Wheat bran)) OR (Soy polysaccharides)) OR (Lignin)) OR (Resistant starch))) AND ((((((((((Diarrhea) OR (Diarrhoea)) OR (diarrheal)) OR (Bowel movement)) OR (Stool)) OR (Feces)) OR (Fecal)) OR (Stool form)) OR (Stool frequency)) OR (Stool frequency)) |
| 50 | ((((((((Tube-fed) OR (Tube feeding)) OR (Enteral nutrition)) OR (Enteral feeding)) OR (Enteral alimentation)) OR (Nutritional support)) OR (Nutrition therapy)) AND (((((((((((((Fiber) OR (Fiber-enriched)) OR (Fibre)) OR (inulin)) OR (Psyllium)) OR (FOS)) OR (Fructo-oligosaccharides)) OR (Oligofructose)) OR (oligosaccharides)) OR (Wheat bran)) OR (Soy polysaccharides)) OR (Lignin)) OR (Resistant starch))) AND ((((((((((Diarrhea) OR (Diarrhoea)) OR (diarrheal)) OR (Bowel movement)) OR (Stool)) OR (Feces)) OR (Fecal)) OR (Stool form)) OR (Stool frequency)) OR (Stool frequency)) |
| 51 | ((((((((Tube-fed) OR (Tube feeding)) OR (Enteral nutrition)) OR (Enteral feeding)) OR (Enteral alimentation)) OR (Nutritional support)) OR (Nutrition therapy)) AND (((((((((((((Fiber) OR (Fiber-enriched)) OR (Fibre)) OR (inulin)) OR (Psyllium)) OR (FOS)) OR (Fructo-oligosaccharides)) OR (Oligofructose)) OR (oligosaccharides)) OR (Wheat bran)) OR (Soy polysaccharides)) OR (Lignin)) OR (Resistant starch))) AND ((((((((((Diarrhea) OR (Diarrhoea)) OR (diarrheal)) OR (Bowel movement)) OR (Stool)) OR (Feces)) OR (Fecal)) OR (Stool form)) OR (Stool frequency)) OR (Stool frequency)) |
| 52 | ((((((((Tube-fed) OR (Tube feeding)) OR (Enteral nutrition)) OR (Enteral feeding)) OR (Enteral alimentation)) OR (Nutritional support)) OR (Nutrition therapy)) AND (((((((((((((Fiber) OR (Fiber-enriched)) OR (Fibre)) OR (inulin)) OR (Psyllium)) OR (FOS)) OR (Fructo-oligosaccharides)) OR (Oligofructose)) OR (oligosaccharides)) OR (Wheat bran)) OR (Soy polysaccharides)) OR (Lignin)) OR (Resistant starch))) AND ((((((((((Diarrhea) OR (Diarrhoea)) OR (diarrheal)) OR (Bowel movement)) OR (Stool)) OR (Feces)) OR (Fecal)) OR (Stool form)) OR (Stool frequency)) OR (Stool frequency)) |

**Supplementary Table S2:** Excluded full texts with reasons

| **No** | **Authors** | **Year** | **Title** | **Journal** | **DOI** | **Reason** |
| --- | --- | --- | --- | --- | --- | --- |
| 1 | Bass, D. J. et al | 1996 | The effect of dietary fiber in tube-fed elderly patients | J Gerontol Nurs | 10.3928/0098-9134-19961001-14 | Not RCT, retrospective study |
| 2 | Nakao, M. et al | 2002 | Usefulness of soluble dietary fiber for the treatment of diarrhea during enteral nutrition in elderly patients | Nutrition | 10.1016/s0899-9007(01)00715-8 | Not RCT |
| 3 | Shimoni, Z. et al | 2007 | The addition of fiber and the use of continuous infusion decrease the incidence of diarrhea in elderly tube-fed patients in medical wards of a general regional hospital: a controlled clinical trial | J Clin Gastroenterol | 10.1097/01.mcg.0000225662.23179.b6 | Not RCT |
| 4 | Khalil, L. et al | 1998 | The Effect of Enteral Fibre-Containing Feeds on Stool Parameters in the Post-Surgical Period | Singapore Medical Journal | PMID: 9676145 | Did not report number of patients who developed diarrhea. |
| 5 | Rayes, N. et al | 2002 | Early enteral supply of fiber and Lactobacilli versus conventional nutrition: a controlled trial in patients with major abdominal surgery | Nutrition | 10.1016/s0899-9007(02)00811-0 | No diarrhea outcome |
| 6 | Rushdi, T. A. et al | 2004 | Control of diarrhea by fiber-enriched diet in ICU patients on enteral nutrition: a prospective randomized controlled trial | Clin Nutr | 10.1016/j.clnu.2004.04.008 | Did not report number of patients who developed diarrhea. |
| 7 | Shankardass, K. et al | 1990 | Bowel function of long-term tube-fed patients consuming formulae with and without dietary fiber | JPEN J Parenter Enteral Nutr | 10.1177/0148607190014005508 | Did not report number of patients who developed diarrhea.  Reported outcome as stool frequency. |
| 8 | Vandewoude, M. F. et al | 2005 | Fibre-supplemented tube feeding in the hospitalised elderly | Age Ageing | 10.1093/ageing/afh242 | Not clear about diarrhea outcome |
| 9 | Ohkura, Y. et al | 2019 | Randomized controlled trial on efficacy of oligomeric formula (HINE E-GEL(R)) versus polymeric formula (MEIN(R)) enteral nutrition after esophagectomy for esophageal cancer with gastric tube reconstruction | Dis Esophagus | 10.1093/dote/doy084 | Both arms received fiber in EN |
| 10 | Schmidt, S. B. et al | 2019 | The effect of a natural food based tube feeding in minimizing diarrhea in critically ill neurological patients | Clin Nutr | 10.1016/j.clnu.2018.01.007 | Both arms received fiber in EN |
| 11 | Emery, E. A. et al | 1997 | Banana flakes control diarrhea in enterally fed patients | Nutr Clin Pract | 10.1177/011542659701200272 | Fiber as a treatment of diarrhea, not prevention |
| 12 | Homann, H. H. et al | 2004 | The beneficial effects of PHGG in enteral nutrition in medical and surgical patients | Clinical nutrition | 10.1016/j.clnu.2004.09.009 | Duplicated report of study |
| 13 | D’Onofrio, V. et al | 2021 | Effects of a Synbiotic Formula on Functional Bowel Disorders and Gut Microbiota Profile during Long-Term Home Enteral Nutrition (LTHEN): A Pilot Study | Nutrients | 10.3390/nu13010087 | home enteral not hospitalized |
| 14 | Rosli, D. et al | 2021 | Randomized Controlled Trial on the Effect of Partially Hydrolyzed Guar Gum Supplementation on Diarrhea Frequency and Gut Microbiome Count Among Pelvic Radiation Patients | JPEN | 10.1002/jpen.1987 | Not tube feeding |


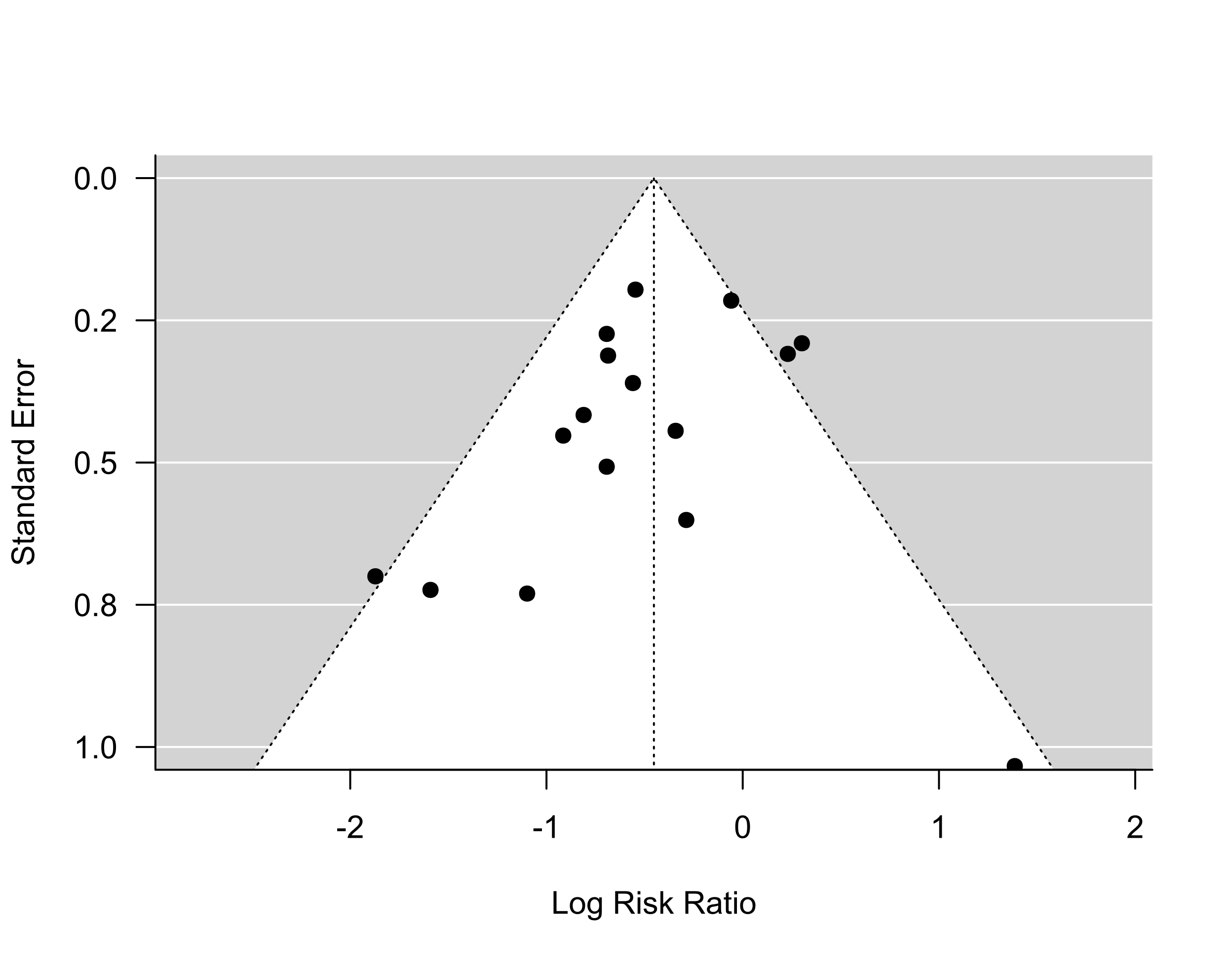


**Supplementary Figure S1**: Funnel plot of 16 RCTs (Egger’s test p-value 0.216)
